# Family Physicians’ Feedback on the Feature Design of a Digital Health Platform to Streamline the Care of Older Adults

**DOI:** 10.3390/geriatrics9060154

**Published:** 2024-11-28

**Authors:** Marjan Abbasi, Sheny Khera, Julia Dabravolskaj, Amira Aissiou, Reza Abbasi-Dezfouly

**Affiliations:** Department of Family Medicine, University of Alberta, Edmonton, AB T6G 2T4, Canada

**Keywords:** older adults, geriatrics, primary care, eHealth, healthcare technology

## Abstract

**Background/Objectives**: Family physicians are essential to a well-functioning healthcare system; however, they face significant administrative and cognitive burdens that contribute to their burnout and reduce the quality of patient care they provide. Digital health tools offer potential solutions to these problems. This study examined the interface design and features of a digital health platform, Carmi, designed to mitigate administrative inefficiencies and cognitive overload by asynchronous patient data gathering and automated report generation. **Methods**: We conducted semi-structured interviews with nine family physicians practicing in Alberta, Canada, to gather their feedback on Carmi’s interface design and features. Participants were asked to view a 20 min virtual demonstration of Carmi and provide input on its interface, navigation, potential impact on their clinic workflow, and suggestions for additional features. Interviews were transcribed and thematically analyzed using NVivo. **Results**: Participants found Carmi’s interface user-friendly; most agreed that Carmi could reduce cognitive burden by automatically generating summary reports of assessments completed by patients and facilitating care coordination. Participants thought integration within existing electronic medical records was important, albeit Care of the Elderly physicians saw the value of Carmi as a standalone platform, noting that it can become a collaborative space where all healthcare providers can contribute to patient care. **Conclusions:** Carmi has the potential to improve primary care efficiency, especially for older adults with complex health needs. Work is underway at several pilot sites that have implemented Carmi so far to gather physicians, patients, and their caregivers’ feedback on its usability.

## 1. Introduction

Family physicians play an essential part in a well-functioning healthcare system; however, they face significant challenges that threaten their professional well-being and the quality of patient care they provide [1]. A recently published report by the Canadian Medical Association showed that more than 50% of physicians experience anxiety and depressive symptoms, and high emotional exhaustion [2]. At least in part, this high prevalence of mental health issues and symptoms of burnout can be attributed to the overwhelming administrative workload that physicians deal with on a daily basis: one in two respondents reported having marginal or poor control over workload, and a third of the respondents described the atmosphere in their primary work area as chaotic [2]. Almost 60% of physicians reported that their electronic medical records (EMRs)—i.e., a digital version of paper charts used in their clinical practice—add to the frustration of their day [2]. While EMRs are considered an internal organizational system, electronic health records (EHRs) are defined as an inter-organizational system [3]. Whether EMRs or EHRs, evidence is emerging that these digital health records, which were initially designed to improve efficiency and patient care, seem to add to, rather than decrease, cognitive burden on physicians [4]. This cognitive burden is attributed to the cognitive effort required to understand medical terminology and interpret test results to make clinical decisions (i.e., intrinsic load); navigate the complex interface of existing digital health records with multiple tabs and menus, and manage notifications and alerts (i.e., extraneous load); and synthesize data from various sections to fully understand each patient’s health conditions and needs and develop an individualized treatment plan (i.e., germane load) [4]. The existing literature points to complex user interfaces and inadequate ways of presenting large amounts of clinical data to physicians as being the two greatest contributors to cognitive burden and rising burnout rates [4], which have been linked to decreased motivation and morale [5].

The issue is further exacerbated by the complexity of patient care, which will only increase as older adults are a rapidly growing demographic group projected to comprise 23% of the population by 2030 and reach 10.4 million by 2037 [6]. Patient care in this age group often requires managing multiple chronic conditions and polypharmacy, as well as addressing unique psychosocial needs [7]. There is an urgent need to support family physicians by optimizing their workflow [8]. Digital health tools, powered by the rapid advancements in the field of artificial intelligence (AI) and large language models (LLMs) [9], have the potential to improve efficiencies in patient care while also reducing administrative and cognitive burdens on physicians. There are early signs that the uptake of these technologies may be high; over 80% of physicians believe that the right technology can greatly reduce their administrative burdens, and 67% expect it to help decrease their stress levels [10]. While these results are encouraging, new technology should prioritize improving the user interface, simplifying information, and reducing documentation time to minimize cognitive burden and encourage widespread adoption [4]. The authors (MA, SK) integrated these strategies, along with their expertise in geriatric care (i.e., development of a nationally awarded integrated team-based geriatric program [11]) and ongoing collaboration with professionals across diverse healthcare settings, into the development of Carmi (https://www.carmahealth.net/). Unlike the existing platforms in the Canadian digital health landscape, this digital health platform integrates asynchronous patient health assessments, advanced decision-making tools, and care coordination with the ultimate goal of optimizing care while easing physicians’ cognitive and administrative burdens. Initially tailored to manage the complex care needs of older adults, Carmi has the potential to be successfully adopted by clinical practices that serve broader populations.

By providing tailored action plans, personalized care management, and curated resources, Carmi is poised to improve care delivery for older adults. However, while digital health solutions present opportunities to enhance efficiency, reduce administrative burdens, and improve patient care quality [12], the adoption of these digital products at scale largely depends on their usability, which should be rigorously evaluated [13]. In this qualitative study, we recruited nine family physicians to view a virtual demonstration of Carmi and provide their feedback on its user interface, flow, and design. The overarching objective of this study was to gather participants’ feedback to refine and optimize Carmi for future scale-up to primary care practices.

## 2. Materials and Methods

### 2.1. Carmi: Description of the Digital Health Solution

**Figure 1:**
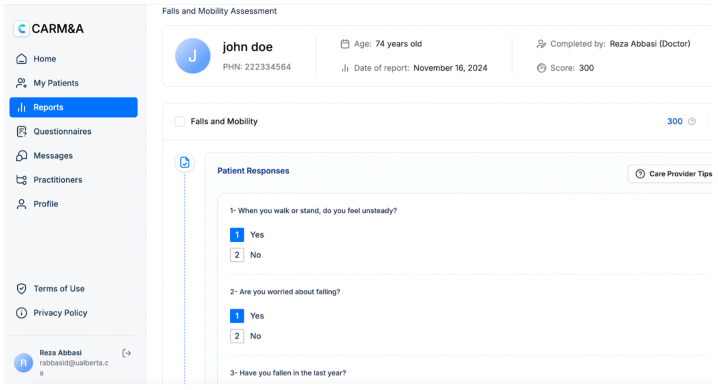
An excerpt from the vitality assessment (i.e., Fall and Mobility Assessment).

Carmi is a cloud-based, AI-augmented digital health platform with three core components: (1) Carmi Care Coordination Hub serves as a shared platform that facilitates communication among healthcare professionals, patients, and caregivers; (2) Assessment Builder enables providers to create customized health assessments that inform personalized care plans; and (3) Carmi Analytics—an AI-augmented feature that automates report generation and provides real-time clinical insights (i.e., provider care tips and links to community resources and services for patients). For the use case of older adults with complex care needs, the Carmi platform enables healthcare providers to deploy the “vitality assessment” to their patients asynchronously from a clinic visit. The vitality assessment is a multidomain assessment based on the principles of the gold-standard comprehensive geriatric assessment [14]. The vitality assessment includes questions related to medical, physical, functional, social, and psycho-cognitive areas of health (see an excerpt in Figure 1) and is designed with older adults in mind; all questions are written in lay language and a large font, and the assessment can be paused if needed.

**Figure 2:**
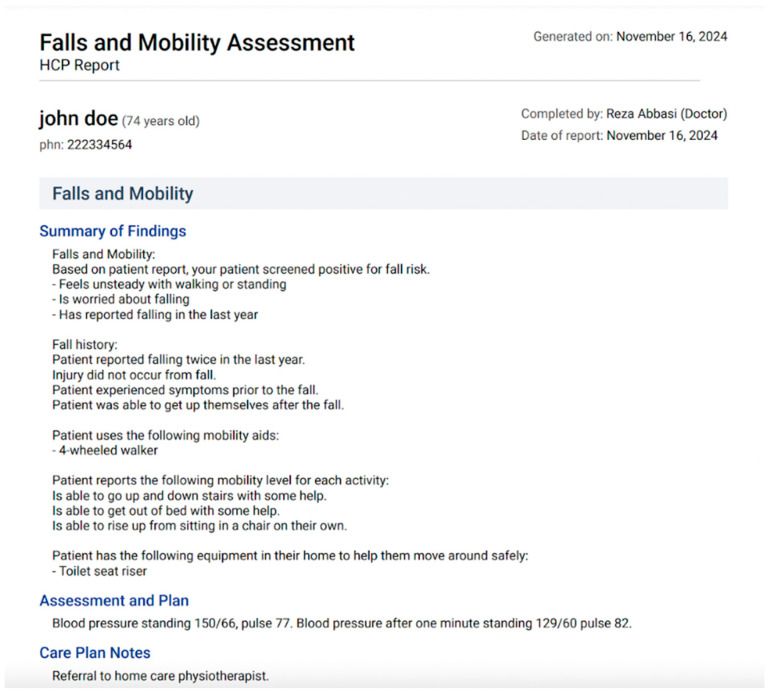
Screenshot of Carmi with mock-up patient data and a summary report based on the falls and mobility section of the completed vitality assessment.

The vitality assessment can be completed in less than 30 min in the comfort of the patient’s home and with caregiver assistance if needed. Based on the vitality assessment results, Carmi generates a summary report using a rule-based recommender system (see an example in Figure 2), which is then sent back to the physician along with the vitality assessment.

**Figure 3:**
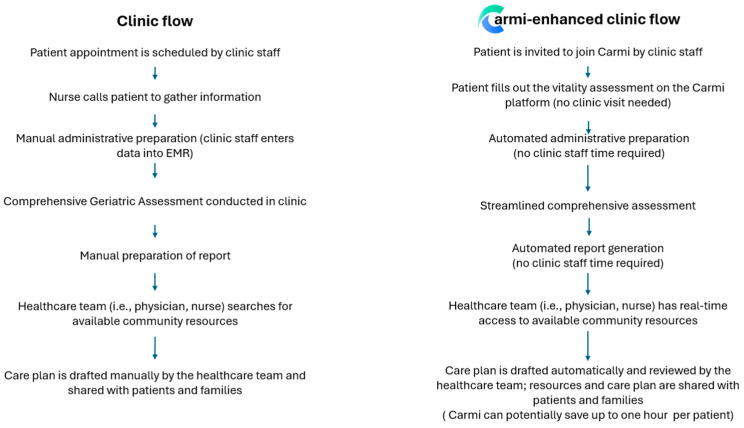
Existing clinic flow vs. Carmi-enhanced clinic flow.

Carmi provides physicians with links to existing community services and resources relevant to the conditions flagged in the vitality assessment. The finalized care plan can be printed or transferred electronically to the physician’s EMR system. Importantly, physicians can invite other healthcare professionals involved in patient care to Carmi, thus facilitating collaboration between members of the care team and streamlining patient care. See the flowchart of the clinical workflow with and without Carmi in Figure 3.

### 2.2. Sampling and Recruitment

The study was conducted between April and July 2024. We recruited a purposive sample of family physicians practicing in Edmonton (Alberta, Canada) and surrounding small towns who—at the time of the study—had an active practice (fee-for-service or non-fee-for-service) and served a diverse patient panel, including older adults (65 years and older). In April and May 2024, prospective participants were identified through the personal network of two physicians on the study team (MA, SK). We decided to leverage MA and SK’s personal network since it represents a diverse pool of physicians, including former residents who have recently entered practice, as well as experienced physicians with established careers and those practicing in urban and rural areas. Moreover, physicians can at times be difficult to recruit for research due to their demanding schedules. Therefore, recruiting within this personal network was a strategic decision that allowed access to a representative sample of physicians willing to share their feedback and thoughts on how Carmi can be improved prior to its deployment at pilot sites. The research coordinator (AA) approached 31 potential participants via email in late May 2024. Given family physicians’ busy schedules, two email reminders were sent to those who did not respond to the initial communication. Fourteen physicians responded to the initial communication; however, four later declined due to lack of time, one did not attend the scheduled interview, and nine family physicians agreed to be interviewed.

### 2.3. Data Collection

Since this research project aimed to gather physicians’ views and opinions of Carmi, we decided to gather these data through qualitative methods. Semi-structured interviews were conducted between mid-June and the end of July. Interviews lasted between 30 min and 1 h and were conducted via Zoom. Before the interviews, all participants were asked to provide verbal consent to participate in the study and give permission for the interview to be audio-recorded. All recordings were carried out using Zoom’s recording function. The research assistant (RAD) walked each participant through Carmi and demonstrated the entire Carmi-enhanced workflow, from logging into the platform to generating the summary report and care plan (see Figure 1 and Figure 2). The demonstration lasted ~20 min, during which participants were encouraged to stop RAD and ask any questions they might have. Then, they were asked a series of questions to gather their views on Carmi’s interface and functionality. The interview guide was designed by JD, MA, and SK, and included questions about the user interface, features, and functionalities that they perceived to be useful and obsolete, how important it is for Carmi to be integrated with EMRs they use in their practice, and how they envision Carmi within their clinic workflow, among others (see Appendix A). All interviews were summarized and reviewed by the team. Data saturation was deemed to have been reached after the sixth interview, as no new information emerged. However, three additional interviews, already scheduled, were conducted to ensure that the information gathered comprehensively addressed the study objectives.

### 2.4. Data Analysis

Anonymized transcripts were analyzed thematically to identify the common themes surrounding physicians’ perceptions of Carmi’s interface and functionality. Thematic analysis followed the guidelines outlined by Braun and Clarke [15]. The inductive approach was used to allow themes to ‘emerge’ directly from the data, as opposed to trying to fit the data into pre-existing hypothesis or coding frameworks, and included the following steps: (1) familiarization with the data; (2) initial coding to identify broader themes; (3) searching for themes and grouping them based on connections and similarities; (4) reviewing and refining themes to ensure they represent the data; (5) and defining and naming themes. The NVivo 15 software (Denver, CO, USA) was used to facilitate data management and coding.

### 2.5. Ethical Considerations

The University of Alberta Health Research Ethics Board approved the study’s procedures (Pro00129267).

## 3. Results

The sample included nine family physicians, four with extensive experience in geriatrics (i.e., Care of the Elderly [CoE] physicians) (see Table 1). Seven participants currently work in primary care clinics in a large population center (population of 100,000 or more), while two participants have practices in small population centers (population of 29,999 or less). Three participants are between 30 and 39 years old, four are between 40 and 49, and two are 60 or older. Most of the participants have been practicing for at least six years.

### 3.1. Current eHealth Context: “Nothing Is Collated for You, Nothing Is Summarized for You.”

To contextualize the findings in this study, it is important to understand the current eHealth context in Alberta, albeit this is likely true for other provinces and territories in Canada. As one participant noted, the EHR currently implemented across hospitals is cumbersome: *“You have to just keep scrolling. Then [click] ‘Show more,’ then keep scrolling. You know, nothing is collated for you, nothing is summarized for you”* (Participant 3). While this participant noted that the EHR is comprehensive and has advantages (e.g., information is well-organized into relevant categories, such as lab results, diagnostic imaging, visit notes), it appears to lack the functionality valued by healthcare providers. For example, it does not have a built-in or easily accessible inventory of community resources that physicians can tap into when creating care plans. Two broad themes emerged in the interviews: (1) Carmi as a potential solution to existing problems, and (2) future adaptations to increase Carmi’s acceptability and usability (for selected quotes, see Table 2).

### 3.2. Carmi as a Potential Solution: “I’m Happy to See That People Are Working on This Type of Things to Try and Solve Problems in Primary Care.”

All participants highlighted that, from the physician’s perspective, Carmi appears user-friendly, partly because there is a good balance between clicking, toggling, and typing, which reduces cognitive load and minimizes ‘clicking fatigue.’ Participants appreciated the ease of adding other healthcare providers to Carmi, thus making it a collaborative space that all allied healthcare providers involved in patient care can contribute to and gather information from. Carmi’s ability to automatically generate a summary report based on the vitality assessment filled out by patients and/or caregivers was perceived by participants as a major benefit of this platform over existing ones. Half of the participants appreciated the adaptive nature of the vitality assessment, meaning that follow-up questions appear only if a health deficit or issue is present, thus alleviating the burden on patients and/or caregivers and ensuring that the summary report contains only the pertinent information. The automatic report generation was appreciated by most participants because of its perceived potential “*to reduce [their] workload*” and “*improve the patient care efficiency*” (Participant 9), which would ultimately free up more time to spend with patients and discuss the specifics of the care plan. Participants noted that summary reports were concise, easy to read, and well-organized.

Carmi particularly appealed to participants who serve a more complex and frail patient population and those who do not follow the fee-for-service model (i.e., family physicians in academic clinics paid by an alternative relationship plan) since their income does not depend on the number of visits per day and therefore there is no pressure to keep visits short. All participants noted that Carmi can be easily integrated into the clinic workflow if medical office assistants (MOAs), nurses, or other clinic staff take on the responsibility of registering patients, sending questionnaires to them and/or their caregivers, and making sure the care plan is uploaded to the patient’s EMR. While these administrative tasks can be offloaded to the support staff, participants agreed that the vitality assessment should be tied to a clinic visit: after physicians review the vitality assessment and/or summary report, patients should be brought in to discuss the findings and care plan. However, some participants noted that the need for a clinic visit may disappear as more practices shift toward virtual care.

### 3.3. Future Adaptations to Increase Carmi’s Acceptability and Usability

Some participants commented that while they—as physicians—found Carmi very user-friendly, patients may encounter difficulties due to the lack of computer literacy. Therefore, they might need to rely on their caregivers to help access and fill out the vitality assessment, although *“that may change over the next 20 years”* (Participant 6). The digital divide is not only generational: Participant 7 noted that some even younger seniors who live in rural areas *“do not know how to work with a smartphone, or they [do not] have an email address or even a laptop. […] Most of the people are farmers, so they still prefer old paperwork”*. While the digital divide likely presents fewer challenges to patients with informal caregivers, it certainly can be more difficult to circumvent for those patients who rely on formal (i.e., paid) caregivers as they usually provide care to several seniors and have limited availability. However, as Participant 5 noted, tackling the digital divide *“is not something in [Carmi’s developers] control”.*

At the moment, Carmi is a standalone cloud-based platform, which might be a barrier to its widespread adoption. Ultimately, integration into EMRs would alleviate the ‘portal fatigue,’ since physicians are often required to navigate multiple platforms, sometimes in the absence of any additional support staff (e.g., MOAs, nurses). Integration into EMRs *“… would make Carmi easy to use. I think, you know, anything that can integrate is always easier. […] although if it’s valuable in its own right, then obviously you want to use it [even if it is a standalone platform]”* (Participant 6). Indeed, participants thought that the likelihood of adopting Carmi as a standalone platform may vary depending on the physician’s practice type and patient demographic. For example, CoE physicians saw value in Carmi, even as a standalone platform. One of these participants mentioned that *“[since] it is its own platform and it’s mobile […] I can easily deploy it wherever I go, instead of waiting to come back to the office to use it”* (Participant 9).

Additionally, some participants expressed hope that the next iteration of Carmi will (1) enable two-way communication between patients and providers, (2) allow physicians to customize the vitality assessment to the specific needs of the patient population they serve and add questions that provide contextual information (e.g., *how* did the patient fall?), (3) enable report generation for selected domains, and (4) display only metrics that are valued by physicians (e.g., number of vitality assessments that are yet to be accessed and completed by patients).

## 4. Discussion

This study represents the first step in the evaluation of Carmi—a digital health platform powered by advanced AI technology and designed to address challenges arising from the growing older adult population and physicians’ administrative and cognitive burdens. In this study, nine family physicians practicing in primary care clinics in large and small population centers in Alberta, Canada, were asked to provide their feedback based on the virtual demonstration of Carmi and its functionality. Overall, participants viewed this platform as a potential solution, which can be valuable to physicians who care for a senior population that is, on average, more frail. Participants commented on both the perceived benefits and challenges with the adoption of the Carmi platform in the primary care setting. The positive aspects included its intuitive design, user-friendliness, the adaptive and patient-centric nature of the vitality assessment, and its utility as a shared digital space for healthcare teams to co-design a care plan with their patients. Participants also suggested modifications that could result in Carmi’s adoption by a greater number of primary care practices, i.e., integrating Carmi into existing EMRs, enabling two-way communication between patients and physicians, allowing customization of the vitality assessment on the physician’s end, generating reports for separate domains of the vitality assessment, and revising metrics displayed on the physician’s dashboard to include only those that are likely to be more informative (e.g., the number of assessments that have not been completed yet).

Carmi’s ability to facilitate patient assessments outside of clinic time (i.e., asynchronously) was perceived to improve patient access to care and alleviate the pressure of clinic-based time constraints, thereby reducing providers’ administrative burden. Furthermore, the automatic generation of summary reports directly from patients’ vitality assessments was highly desired. Participants noted that this core functionality (which was informed by the principles of user-centered design) has a great potential to decrease documentation time, thus allowing physicians to focus more on patient care rather than administrative tasks.

Another notable benefit highlighted by participants was the cognitive support provided by an easy access to the inventory of available community resources that are relevant to health conditions flagged in the vitality assessment. By presenting evidence-informed guidelines, pathways, and tailored resources, the tool empowers healthcare providers in their decision-making processes at the point of care. This support not only enhances clinical decision-making but also helps mitigate cognitive overload, particularly when managing patients with complex needs. Moreover, healthcare providers can seamlessly send completed care plans to patients and caregivers, thereby closing a communication gap in the current healthcare system and fostering patient agency and engagement. Even though the Supreme Court of Canada acknowledged in 1993 that patient information belongs to the patient, in fact, the physical record belongs to the organization that created it [17]. Therefore, until recently, Canadians had no or very limited access to their health information, even despite the evidence that 88% of respondents reported feeling “more informed about [their] health as a result of accessing [their] personal health information online” and 82% reported that they “can better manage [their] health because [they] can access [their] personal health information online” [18].

While the Carmi platform was well-received by the family physicians who participated in this study, they noted some limitations in the demonstrated version of Carmi. Most notably, its lack of integration with existing EMRs, which can hinder workflow efficiency and contribute to ‘portal fatigue’. Despite an estimated 93% of Canadian primary care physicians reporting using EMRs in their clinical practices in 2022 [19], there is no singular EMR. Instead, each primary care practice chooses which EMR vendor to procure. In Alberta, there are several large and small vendors (e.g., Telus Health, Microquest, WELL Health, QHR Technologies) that offer an array of EMRs [20] with different priorities, features, and technical abilities to integrate with other digital health applications. Reaching an agreement with these vendors and achieving integration will require collaborative efforts between developers, healthcare organizations, and policy-makers. The Canadian government is prioritizing interoperability among digital health systems through legislation (i.e., Bill C-27 Digital Charter Implementation Act, 2022 [21]) and modernizing data privacy and protection frameworks; however, the actualization of seamless data exchange is still a work in progress.

As digital health technologies continue to evolve, ongoing adaptations are required. However, the rapid integration of AI technology into clinical practice comes with a mix of excitement and apprehension within the healthcare community. Nearly two-thirds of physicians express concerns about the implementation and effectiveness of AI in healthcare [22]. Recently, the College of Family Physicians of Canada issued a statement emphasizing the importance of AI tools in family medicine [23]. This statement underscores the necessity for these tools to tackle issues that directly or indirectly affect family practice, to be co-developed with end users, and to support team-based care, among others. Additionally, due to the potential for generative AI to produce inaccurate responses, there is a crucial need for “human-in-the-loop” oversight to ensure the accuracy of report generation. In response to the aforementioned concerns, the next generation of Carmi integrates generative AI [24] and Retrieval-Augmented Generation (RAG) [25] to enhance its efficiency and the quality of report generation; Human-in-the-Loop (HITL) processes to ensure that all AI-generated content is reviewed and approved by clinicians; and advanced Prompt Engineering techniques, such as chain-of-thought prompting and multi-shot learning, to guide generative AI in producing higher-quality outputs tailored to clinical needs.

There are study limitations that deserve attention. First and foremost, this project aimed to gather preliminary feedback on Carmi’s interface and functionality to inform improvements before its implementation at several pilot sites in Canada. Thus, we want to make it clear that this project was the first step in the rigorous and multi-step evaluation of Carmi. In this project, participants provided their feedback based on a 20 min virtual demonstration, rather than real-time engagement. To assess Carmi’s usability, efforts are currently underway at multiple pilot sites that have implemented Carmi so far to gather qualitative (i.e., interviews with physicians, patients, and their caregivers) and quantitative data (i.e., the System Usability Scale [26]). As part of this next phase of Carmi’s evaluation, not only physicians but also patients and caregivers are being asked to reflect on whether the platform is accessible and easy to navigate. Another limitation is that physicians working in small population centers were underrepresented, and they may have more diverse perspectives. Although we reached data saturation by the sixth interview (likely due to this study’s goal to elicit preliminary thoughts and opinions on Carmi’s interface and functionality prior to launching it in several pilot sites), the ongoing evaluation of Carmi’s usability is being conducted in a larger and more diverse sample of physicians, patients, and caregivers.

## 5. Conclusions

Innovative technologies are urgently needed to alleviate the administrative and cognitive burdens faced by physicians and other healthcare providers. However, new technologies should be designed thoughtfully and with key end-users’ feedback; otherwise, they might add to these already significant administrative and cognitive burdens. These considerations were kept front and center during the development of Carmi. In this study, we gathered feedback from nine physicians on its interface and functionality. The platform was deemed user-friendly, with an intuitive design, and was seen as a shared digital space for healthcare teams to collaboratively design care plans with patients. Several modifications were suggested. While some of them (e.g., integrating Carmi into existing EMRs, enabling two-way communication between patients and physicians) are challenging to implement in the next version of Carmi, work is underway to allow for further customization of assessments and improvement of the report generation using generative AI and RAG. The next step in Carmi’s evaluation includes gathering qualitative and quantitative data from healthcare providers, patients, and caregivers who have had a chance to interact with Carmi at several pilot sites.

## Figures and Tables

**Table 1:** Description of the sample of nine family physicians that took part in the study.

Participant ID	Age Range	Location of Practice ^1^	Current Role	Years of Practice in the Current Role
Participant 1	40–49	Large PC	CoE physician	1 to 5 years
Participant 2	30–39	Large PC	Family physician	8 years
Participant 3	60–69	Large PC	CoE physician	Over 20
Participant 4	30–39	Large PC	Family physician	6 years
Participant 5	40–49	Small PC	Family physician	15 to 20 years
Participant 6	40–49	Large PC	Family physician	12 years
Participant 7	40–49	Large PC	Family physician	1 to 5 years
Participant 8	30–39	Large PC	CoE physician	1 to 5 years
Participant 9	60–69	Small PC	CoE physician	Over 20

^1^ As per Statistics Canada [16], a small population center (PC) is defined as a center with a population between 1000 and 29,999; a medium PC has a population of 30,000 to 99,999; and a large PC has a population of 100,000 and more. Abbreviations: CoE, Care of the Elderly; PC, population center.

**Table 2:** Selected quotes pertaining to two main themes.

Code	Quote
Theme 1: Carmi as a potential solution	
User-friendly design	“[Carmi is] laid out quite well: everything is very clearly where you would expect to look for certain tabs or headings.” *Participant 2*
	“I liked that most things were either things that you just have to click or things that you just toggle. So, I thought the balance between that and typing was very appropriate [and allows to] decrease the burden of either task. I know we talked about clicking fatigue, where there’s just so many things to click that it just gets mind boring, but I thought this was done in a very smart way.” *Participant 5*
	“My EMR is Ava, and I find Ava’s setup is kind of similar in that […] it’s been fairly intuitively designed as well. So [Carmi’s design] is familiar to me.” *Participant 6*
	“[Carmi’s design is] to be honest, even more user-friendly, compared to our EMRs and stuff.” *Participant 8*
Collaborative space	“People are looking into the spirit of making documentation and consultations easy so that we can focus on the essentials. And this platform, properly deployed with necessary adjustments, makes it easy to achieve that goal. […] If you need to send the care plan to the social worker—[…] you just click and it sends to the next person who is going to execute that care plan.” *Participant 4*
Automatically generated summary reports	“I certainly love the way the [summary] report was created from the vitality assessment. It was very nice, you know, one page—and you got all that input.” *Participant 3*
Adaptive nature of the vitality assessment	“It gets to ask the patient what you really want to ask. […] So you are able to be selective and very specific and focus on what you want to assess. [This] gives you a better response or a better report of what you’re trying to assess. Instead of having so many things on your report, it narrows you down to the essentials so it’s quite useful in that manner.” *Participant 4*
Well-suited for practices serving more complex patients	“I don’t think every older patient needs [Carmi]. But those that I feel that are more frail, more complex ones, [the ones] I’m worried about… I could see Carmi being an important tool to make sure that I’ve checked all the boxes and done everything that I could. Time is a barrier [more so] for fee-for-service providers.” *Participant 5*
Ease of integration within the clinic workflow	“That’s another thing, because the goal is to reduce the administrative work on the physician. I use different tools. […] And I delegate my MOA ‘Look, I need this patient to fill this out.’ So that by the time they’re finished with that assessment, then I come in to do the clinical aspect of it.” *Participant 4*
**Theme 2: Future adaptations to increase Carmi’s acceptability and usability**	
Integrating Carmi within existing EMRs	“You have like 20 million different things that you have to log into and […] in order to make someone want to use [Carmi], reducing that fatigue and burden is […] important in making sure that people feel good about using it.” *Participant 5* “[EMR integration is] very important, because you know, when you log in into something, and you go logging into something else, a different platform, it’s a little bit complicated and can be challenging […] especially if you don’t have support, like a nurse or someone who can take over and do this on the side. If you want to do it yourself, it is going to be time-consuming for a family physicians, if I’m geriatric specialist—maybe…. But for family physicians, we try, you know, to get into something that’s easy and time efficient.” *Participant 8* “Someone whose community practice is older […] I think that it would be less of a barrier to have Carmi as an extra because they’d be accessing it more often. But for someone who has a younger clinic practice and isn’t seeing the frail elderly in the community as much, it may be a barrier if it’s a separate [application], because I mean… just being realistic… I don’t want to have all sorts of different, you know, apps or whatever, to be signing into.” *Participant 2*
Enabling two-way communication between patients and physicians	“Honestly, you won’t believe how many messages I get now, texts and emails from families, and they’re not overly intrusive or anything, but they want to know or they want to inform me, and I’m texting them back, I’m emailing them. Can you imagine if I had a platform like that? […] Whenever [principal investigators] are ready, you know, to develop a two way communication on the care plan.” *Participant 3*
Enabling customization of the vitality assessment	“If I just want to ask about bowel and bladder [functions], then I can set it up and then just only send that part and the patient just will see that [one] and not the whole questionnaire.” *Participant 1* “For example, you cannot see that this patient has a presence of weakness [and] that’s why he’s falling. There is no room to write that in a questionnaire.” *Participant 4*
Enabling report generation for a selected domain	“Let’s say if we are just asking for some specific parts. The patient has already done the comprehensive assessment, and we have all the information, we just need to generate one piece of the whole part. It would be much easier if we can just do it and generate and send it back to the patient, go on with the care plan, and do the rest over time, rather than just spend so much time for something that we don’t need at this time.” *Participant 1*
Displaying only relevant dashboard metrics	“I’m just trying to think of the reason why you would log in. It would be to check a patient’s response, to generate a report, or something like that. So I mean, I think something related to the reason someone would log in: for example, number of completed assessments, or number of new reports that haven’t been viewed by you, or number of patients that have not yet received their report or something like that.” *Participant 5*

AI: artificial intelligence; EMR: electronic medical record; MOA: medical office assistants.

## Data Availability

The qualitative data supporting the conclusions of this article will be made available by the authors upon request.

## Appendix A. Interview Guide

As you know, you are invited to participate in a study that aims to gain physicians’ perspectives on the digital solution called Carmi that our team created to help family physicians manage care for complex older patients in the primary care setting. More information can be found in the information letter we shared with you earlier. If you have had time to read the information letter, let me know if you have any questions. If not, please feel free to read it now. If you consent to participate in this research project, the interview will take 30 to 60 min, will be audio-recorded, and then transcribed and analyzed. The only reason why we want to audio-record this interview is to make sure we do not miss any important insights from you. The audio recording will be destroyed as soon as it is transcribed. You will not be asked any personal questions, and we will not be collecting any personal identifying information from you. Do you consent to participate in this interview? [the interviewer fills out the Verbal Consent Attestation form if the answer is yes]

- Do you have any questions about this research project before we get started?
- Which tools/software products might you find particularly helpful in managing care for older patients with complex healthcare needs? Which features should these tools have?
- What software products have you previously tried or are actively using to help you take care of your complex patients? What do you like about the software you are currently using? What about the products that you stopped using? Why did you stop using them?
- Does Carmi appear easy to navigate? Please explain which aspects of the navigation you find challenging. What do you think about the ‘flow’ of this platform, beginning from signing into the platform to reviewing the final report?
- What do you think about the user interface design? Probing questions: Did you find its flow intuitive? Were there any specific features or functionalities you found particularly easy and/or useful? Which ones are difficult and/or obsolete?
- Are there features or functionalities that you think are beneficial in addressing the unique requirements of older patients with complex healthcare needs? Which features do you think are not useful?
- Would you prefer the digital health platform to be integrated with the Electronic Medical Records (EMR) platform you use in your practice? If yes, why do you think it is important?
- Which additional features and functionalities do you think would improve this platform’s usability?
- What do you think about the health report generated by the platform? Are there any important pieces that are missing from this report?
- Would you delegate the task of registering patients on this platform to someone else on your care team? If yes, who would that be?
- What is your overall impression of this digital health platform? Is it something you would try in your own practice? Why or why not?

Thank you so much for participating in this project, we appreciate your time and insights!
